# Mutagenesis Studies and Structure-function Relationships for GalNAc/Gal-Specific Lectin from the Sea Mussel *Crenomytilus grayanus*

**DOI:** 10.3390/md16120471

**Published:** 2018-11-27

**Authors:** Svetlana N. Kovalchuk, Nina S. Buinovskaya, Galina N. Likhatskaya, Valery A. Rasskazov, Oksana M. Son, Liudmila A. Tekutyeva, Larissa A. Balabanova

**Affiliations:** 1 Laboratory of Marine Biochemistry, G.B. Elyakov Pacific Institute of Bioorganic Chemistry, Far Eastern Branch, Russian Academy of Science, 159, Stoletya Vladivostoku str., Vladivostok 690022, Russia; s.n.kovalchuk@mail.ru (S.N.K.); ninok1993@mail.ru (N.S.B.); 2 Laboratory of Bioassays and Mechanism of Action of Biologically Active Substances, G.B. Elyakov Pacific Institute of Bioorganic Chemistry, Far Eastern Branch, Russian Academy of Science, 159, Stoletya Vladivostoku str., Vladivostok 690022, Russia; galinlik@piboc.dvo.ru; 3 Innovative Technology Center, School of Economics and Management, Far Eastern Federal University, 8 Sukhanova St., Vladivostok 690090, Russia; oksana_son@bk.ru (O.M.S.); tekuteva.la@dvfu.ru (L.A.T.)

**Keywords:** galactose-specific lectin, *Crenomytilus grayanus*, carbohydrate-binding site, molecular docking, site-specific mutagenesis, carbohydrate-binding activity

## Abstract

The GalNAc/Gal-specific lectin from the sea mussel *Crenomytilus grayanus* (CGL) with anticancer activity represents а novel lectin family with β-trefoil fold. Earlier, the crystal structures of CGL complexes with globotriose, galactose and galactosamine, and mutagenesis studies have revealed that the lectin contained three carbohydrate-binding sites. The ability of CGL to recognize globotriose (Gb3) on the surface of breast cancer cells and bind mucin-type glycoproteins, which are often associated with oncogenic transformation, makes this compound to be perspective as a biosensor for cancer diagnostics. In this study, we describe results on in silico analysis of binding mechanisms of CGL to ligands (galactose, globotriose and mucin) and evaluate the individual contribution of the amino acid residues from carbohydrate-binding sites to CGL activity by site-directed mutagenesis. The alanine substitutions of His37, His129, Glu75, Asp127, His85, Asn27 and Asn119 affect the CGL mucin-binding activity, indicating their importance in the manifestation of lectin activity. It has been found that CGL affinity to ligands depends on their structure, which is determined by the number of hydrogen bonds in the CGL-ligand complexes. The obtained results should be helpful for understanding molecular machinery of CGL functioning and designing a synthetic analog of CGL with enhanced carbohydrate-binding properties.

## 1. Introduction

Lectins are specific carbohydrate-binding proteins, found in animals, plants and microorganisms, and involved in various biological processes including cell adhesion, innate immunity, fertilization, differentiation et al. [1,2,3,4]. First, classifications of lectins were based on the glycan structures, to which they exhibited high affinity [5]. Later, lectins were classified into families on the basis of similarity of amino acid sequences of their carbohydrate recognition domains (C-type lectins, L-, M-, P-, R-, F-type lectins, galectins et al.) [1,2,6]. To date, the amino acid sequences of several hundreds of lectins have been determined, and a number of their three-dimensional structures have been elucidated. Recently, a new lectin classification based on their three-dimensional structures was proposed and 48 lectin families were characterized [7].

In the last two decades, many lectins from marine invertebrates were identified, and their functions in various immune events were demonstrated [3]. Earlier, we reported on a novel GalNAc/Gal-specific lectin from the mussel *Crenomytilus grayanus* (CGL), which did not share sequence homology with known lectins and consisted of three tandem-repeat subdomains with high (up to 73%) sequence identity to each other [8,9]. Three-dimensional structure prediction revealed that CGL adopted a ß-trefoil fold and contained three binding sites including conserved HPY(K)G motifs [9,10], which was later confirmed by X-ray analysis [11,12].

CGL was shown to possess anti-cancer activity through binding globotriose Gb3 [12]. The ability of CGL to recognize Gb3 on the surface of breast cancer cells [12] and bind mucin-type glycoproteins [8,9], which are often associated with oncogenic transformation, makes structural studies highly valuable to discern mechanistic details of its function. In our previous study the role of three conserved HPK(Y)G motifs in hemagglutinating and carbohydrate binding activities of CGL was experimentally shown by site-specific mutagenesis studies [10]. To investigate CGL functions and peculiarities of its molecular organization in more detail, in this study we evaluated the contribution of individual amino acid residues from CGL binding sites into the lectin activity using analysis of recombinant CGL mutants and in silico evaluation of mono- and oligosaccharide structures impacts on CGL binding properties.

## 2. Results

### 2.1. Analysis of CGL Contacts with Galactose/Galactosamine for Mutagenesis

The theoretical model of the spatial structure of lectin CGL was previously constructed by us [10] based on the crystal structure of the lectin MytiLec determined at 1.05 Å resolution (Protein Data Bank accession: PDB 3WMV) [13]. Superimposition of all Cα atoms of obtained CGL model and CGL crystal structure (PDB 5F8S) [12] showed that they were almost completely superimposable (values of the root-mean-square deviation (RMSD) were 0.4 Å). Thus, the predicted structure of the lectin CGL was in good agreement with the experimentally established CGL structure and suitable for in silico mutagenesis and molecular docking studies.

**Figure 1:**
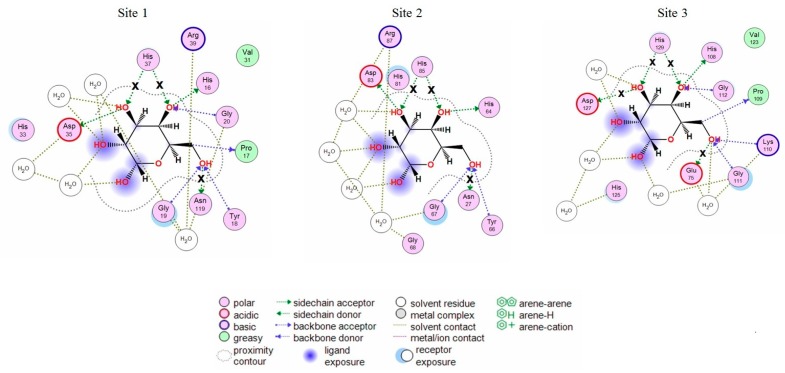
2D-diagrams of the galactose-binding sites (Site 1, 2, 3) in the wild type CGL. Hydrogen bonds lost in the corresponding mutant are indicated with a cross (**x**).

The analysis of CGL contacts with α-galactose (Protein Data Bank accession: PDB 5F8W) and galactosamine (PDB 5F8Y) showed that CGL amino acid residues His37 and Asn119 from Site 1; His85 and Asn27 from Site 2; Asp127, His129, and Glu75 from Site 3 formed hydrogen bonds with these monosaccharides (Figure 1). These residues were selected for mutagenesis experiments.

### 2.2. Mutagenesis Studies

To obtain the recombinant CGL of the wild type and Asn27Ala, His37Ala, Glu75Ala, His85Ala, Asn119Ala, Asp127Ala and His129Ala mutants, expression plasmids were constructed on the basis of pET40/CmAP plasmid described earlier [10,14]. The alkaline phosphatase CmAP in the hybrid CGL-CmAP protein allowed for monitoring recombinant lectins during expression and purification steps [10,14].

**Figure 2:**
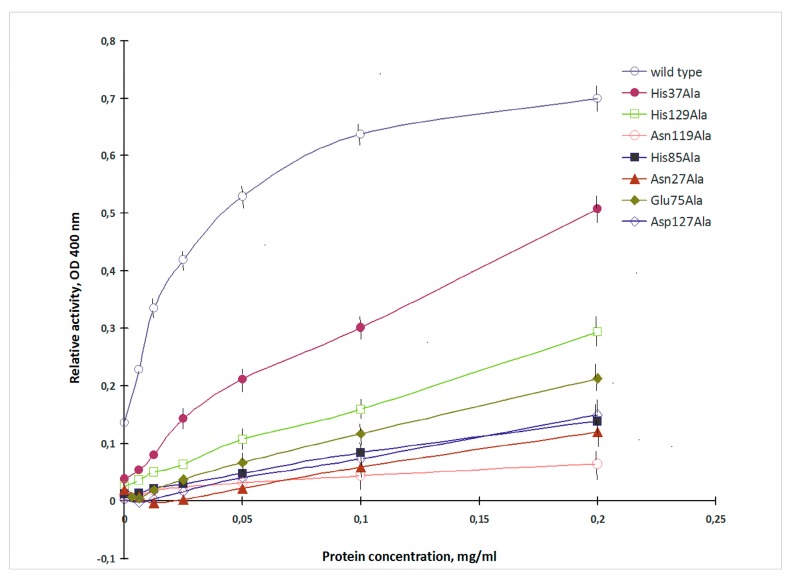
Mucin-binding activity of the wild and mutant types of CGL. The lectin-mucin complexes (axis *X*—mucin concentration) were monitored by measuring the phosphatase activity of CGL/CmAP hybrid (axis *Y*).

CGL was shown to exhibit high affinity to porcine stomach mucin [15]. The mucin-binding activity of the recombinant CGL of the wild and mutant types was evaluated by measuring the alkaline phosphatase activity provided by CmAP domain [10] (Figure 2).

The mucin-binding activity of the obtained mutants varied in a wide range and was from 9% to 73% of the wild lectin (Figure 2). CGL mutants with the alanine substitutions of His37, His129, Glu75, Asp127 and His85, Asn27, Asn119 showed decreased mucin-binding activities in 1.4, 2.3, 3.2, 4.5, 5.0, 5.9 and 11.1 times, respectively.

### 2.3. Analysis of Contacts in Complexes of CGL and Its Mutants with Oligosaccharides

**Figure 3:**
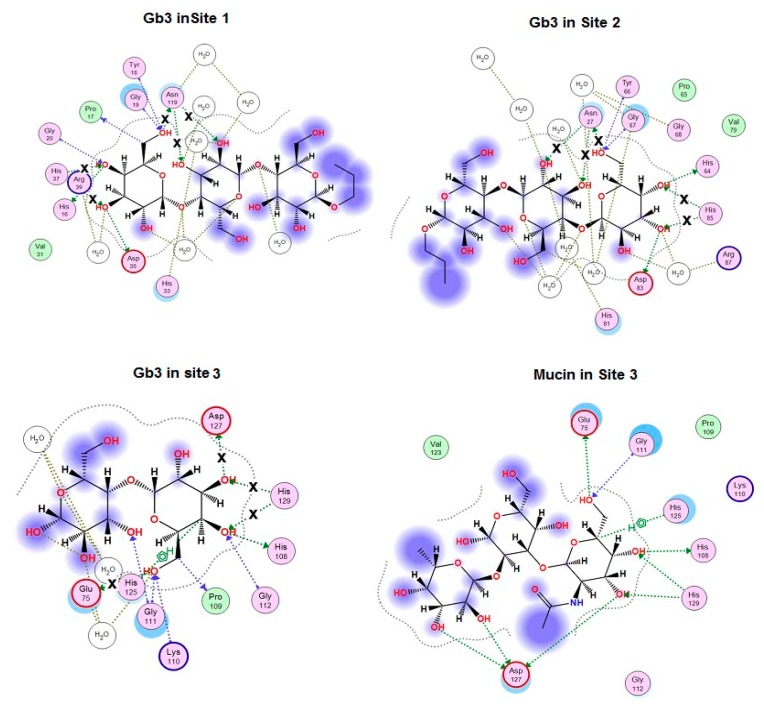
2D-diagram of Gb3-binding sites (Site 1, 2, 3) and mucin binding in Site 3 of the wild type CGL. Hydrogen bonds lost in the corresponding mutant are indicated with a cross (**x**).

It was found that the mucin-binding activities of the obtained mutants did not correlate with changes in the calculated binding energy of galactose with CGL mutants that can be explained by the fact that CGL affinity to different ligands depends on their structure (Table 1). To clarify the impact of the ligand structure on CGL binding activity and binding mechanisms of the attachment of CGL to ligands, in silico analysis of contacts of CGL mutants in complexes with globotriose Gb3 was carried out with MOE 2018.01 program. The obtained results showed that the alanine substitution of His37, His129, Glu75, Asp127, His85, Asn27 and Asn119 residues changed CGL contacts with Gb3 (Figure 3) and the total binding energy of CGL with ligands (Table 1).

The analysis of contacts between CGL and Gb3 has shown that Asn27 and Asn119 residues formed the hydrogen bond not only with C6-OH group of Gb3 terminal galactose residue, but also with the neighboring galactose residue (Figure 3).

It was found that Asn27Ala and Asn119Ala mutants lost three hydrogen bonds with Gb3 in Sites 1 and 2 in comparison with the wild type CGL (Figure 3), what correlates with a drastic decrease in their affinity towards mucin that has two terminal galactose as Gb3 (Table 1, Figure 2).

The residue Glu75 in CGL Site 3 is located in the same position as Asn27 and Asn119 from Sites 1 and 2 and forms the hydrogen bond with C6-OH group of terminal monosaccharide residue similarly to Asn27 and Asn119. According to the modeling results, the mutant Glu75Ala lost only one hydrogen bond (Figure 3) and therefore retained a higher percentage (31%) of the lectin activity than Asn27Ala and Asn119Ala mutants (Table 1).

The His37, His85 and His129 residues form two hydrogen bonds only with the terminal Gb3 monosaccharide residue and the binding energy of His37Ala, His85Ala and His129Ala mutants with both galactose and globotriose are similar (Table 1, Figure 3). However, the lectin activities of the mutants His37Ala, His85Ala and His129Ala were different (Table 1, Figure 2). Apparently, the activities of these CGL mutants depend also on the structural rearrangement of the sites after alanine substitutions of His37, His85 and His129. Distinctive affinities of Sites 1–3 of CGL toward galactose were also shown by NMR titrations [12].

According to the modeling data, Asp127 forms only one hydrogen bond with the terminal monosaccharide of Gb3 (Figure 3). These results fully coincided with crystallographic data from Protein Data Bank (PDB accession numbers: 5F8W, 5F8Y and 5F90). However, the mutant Asp127Ala activity with the use of porcine stomach mucin (PSM) as ligand was only 22% compared to the wild lectin although only one hydrogen bond disappeared in the complexes with galactose and globotriose (Table 1, Figure 3).

**Figure 4:**
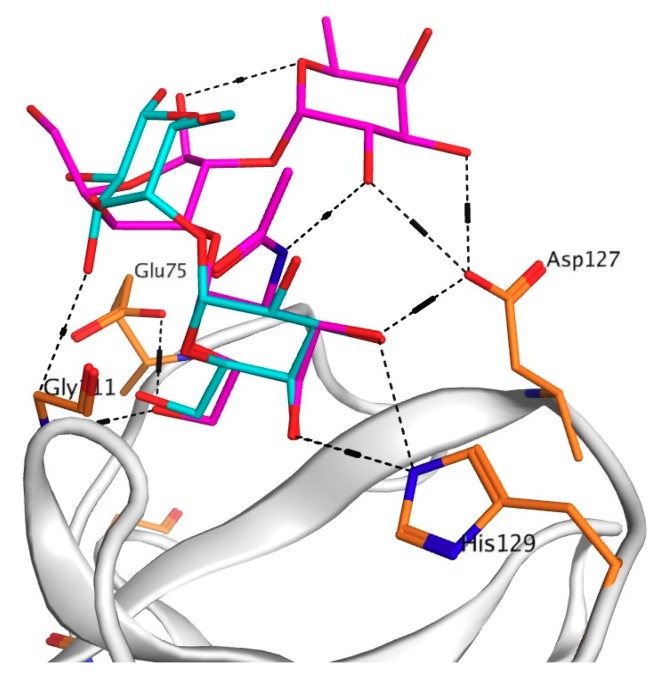
3D-superimposition of globotriose (Gb3) and porcine stomach mucine (PSM) trisaccharides in the binding Site 3 of the wild-type CGL. The structures of the ligands are shown as stick in blue (Gb3) and in pink (PSM).

To explain the drastic change in the activity of this mutant, a model of the mutant Asp127Ala complex with the PSM oligosaccharide was constructed using molecular docking of CGL with the PSM-like trisaccharide of the blood group A epitope GalNAcα1-3Gal [Fucα1-2] since data concerning crystal structure of PSM itself were not available in literature (Figure 3 and Figure 4).

The analysis of contacts between Site 3 of CGL and the PSM-trisaccharide GalNAcα1-3Gal [Fucα1-2] has shown that Asp127 forms a hydrogen bond with C3-OH group of the terminal monosaccharide galactose and two additional hydrogen bonds with OH groups at C2 and C3 of the third residue fucose (Figure 3 and Figure 4). Asp127Ala mutant lost all three hydrogen bonds with the PSM trisaccharide, which can explain a sharp decrease (down to 22% of the wild type CGL) in the mucin-binding activity of Asp127Ala mutant (Table 1, Figure 2).

Asp35 and Asp83 residues in the binding Sites 1 and 2 are located in the same positions as Asp127 in Site 3 and can form three hydrogen bonds with the PSM trisaccharide. The activities of Asp35Ala and Asp83Ala mutants have not been yet studied experimentally, but it may be assumed those will decreased like the case of Asp127Ala mutant.

## 3. Discussion

CGL is the GalNAc/Gal- and mucin-specific lectin with an amino acid sequence that distinguishes it from lectins of known families [9]. To date, this new lectin family which was proposed to name mytilectin [13] includes, besides CGL, five lectins from the sea mussels *Mytilus trossulus* (MTL), *Mytilus galloprovincialis* (MytiLecs 1–3) and *Mytilus californianus* (MCL) [15,16,17,18,19]. These lectins share common ß-trefoil fold and contain three carbohydrate-binding sites [9,10,11,12,13,19]. A β-trefoil fold was proposed for the first time for the crystal structure of Kunitz soybean tripsin inhibitor [20]. Now, it is known that β-trefoil fold is shared by proteins from several subfamilies, including cytokines, ricin B-like lectins, agglutinins, actin-cross-linking proteins etc. [21], which have no sequence similarity and have distinctive ligands, modes of ligand binding and functions.

According to the crystal data, CGL exhibits a characteristic pseudo three-fold symmetry and contains three structurally conserved subdomains [11,12]. Each of these subdomains is composed of four β-strands. Two strands from each subdomain collectively form a six-stranded β-barrel and the remaining two β-strands from each subdomain together form a β-hairpin triplet that caps one end of the barrel [11]. The putative glycan-binding pocket in the first CGL subdomain is formed by the side chains of His16, Tyr18, Val31, His33, Asp35, His37 and Arg39, and the backbone of Gly19 and Gly20 (HYGGVHDHR). The second binding pocket of CGL is formed by the same amino acid residues (HYGGVHDHR). Whereas in the third pocket of CGL, tyrosine is substituted by lysine, and arginine is replaced by alanine (HKGGVHDHA) [11]. The structure of the CGL-galactosamine complex obtained by Liao et al. [12] also revealed in CGL three carbohydrate-binding sites: Site 1 consisted of His16, Gly19, Asp35, His37 and Asn119; Site 2 included His64, Gly67, Asp83, His85, and Asn27; Site 3 comprised His108, Gly111, Asp127, His129, and Glu75. Superimposition of the three carbohydrate binding sites indicates that all three sites contain the same amino acid compositions except for the replacement of Asn for Glu in Site 3. These data confirmed our predications based on homology modeling [10].

In our previous study, we evaluated the contribution of three conserved HPK(Y)G motifs in hemagglutinating and carbohydrate binding activities of CGL by site-specific mutagenesis [10]. According to the obtained data, alanine substitutions of His16, Pro17, Gly19 of Site 1 and His64, Pro65 and Gly67 in Site 2 resulted in complete loss of the CGL hemagglutinating and mucin-binding activities, whereas the mutant CGL with His108Ala, Pro109Ala and Gly111Ala mutations in the Site 3 kept the binding activity against mucin [10].

In this study, we applied the same approach to elucidate the individual contribution of the amino acid residues from CGL binding Sites 1–3 to the carbohydrate binding activity. It was found that the alanine substitution of none of the studied amino acid residues (His37 and Asn119 from Site 1; His85 and Asn27 from Site 2; Asp127, His129, and Glu75 from Site 3) did not lead to the complete loss of the mucin-binding activity of CGL due to the presence of two other normal Sites. But the contribution of these amino acid residues to the mucin-binding activity of CGL was not the same. The replacements of Asn119Ala in Site 1 and Asn27Ala in Site 2 were found to lead to the greater decreasing of the mucin-binding activity of CGL (up to 9% and 17%, respectively) in comparison with the alanine substitution of Glu75 located in Site 3 in the same position as Asn119 and Asn27 from Sites 1 and 2, respectively (Table 1). This confirmed the suggestion of Jakób with co-authors [11] about differences in the affinity (or specificity) for glycan moieties between binding sites and with our previous experimental data [10].

Moreover, in silico analysis of the CGL binding to galactose, globotriose and mucin have shown that the affinity of CGL to these ligands depends on their structures, which determine the number of hydrogen bonds in the CGL-ligand complex and, consequently, its binding energy in total. The maximal decrease in the mucin-binding activity observed for the mutants Asn119Ala in Site 1 and Asn27Ala in Site 2 could be explained by the loss of all three hydrogen bonds with two terminal galactose residues of oligosaccharides in comparison with the wild-type CGL (Table 1, Figure 3). The amino acid residue Asp127 in Site 3 (and similar residues Asp35 and Asp83 in Sites 1 and 2) was found to play a decisive role in the higher lectin specificity to mucin than globotriose (Figure 4). Thus, the efficiency of CGL binding depends on the composition of terminal monosaccharide units in oligosaccharides due to the different capability of CGL amino acid residues from Site 1–3 to bond with OH-groups of the second galactose and third fucose in the addition to the binding with the terminal galactose.

## 4. Materials and Methods

### 4.1. In Silico Analysis of Contacts between CGL and Ligands and Mutagenesis

The model of CGL spatial structure was constructed as described previously [10] on the basis of the crystal structure of the lectin MytiLec established with a resolution of 1.05 Å (PDB code 3WMV) [13]. The analysis of contacts between CGL and ligands, in silico mutagenesis, molecular docking and visualization of the results were carried out with the Ligand interaction and Dock modules of MOE 2018.01 program [22]. The crystal structure of CGL complexes with galactose (PDB 5F8W), galactosamine (PDB 5F8Y), globotriose Gb3 (PDB 5F90) and trisaccharide motif GalNAcα1-3Gal [Fucα1-2] from porcine stomach mucin (PSM-trisaccharide), which is identical with terminal trisaccharide of the blood group A human histo-blood group antigen (HBGA A-trisaccharide) (PDB 2WMI) [23], were used in docking analysis. Molecular docking of PSM-trisaccharide GalNAcα1-3Gal [Fucα1-2] with CGL was carried out using complex with galactosamine (PDB 5F8Y) as a template. The ligand binding energy (the molecular mechanics generalized Born interaction energy) was the non-bonded interaction energy between the receptor and the ligand and comprised van der Waals, Coulomb and generalized Born implicit solvent interaction energies [24]. The change in the binding energy of the CGL mutants with galactose or globotriose was calculated as ΔE = Emut-Ewt. The results were obtained with the use of IACP FEB RAS Shared Resource Center “Far Eastern Computing Resource” equipment (https://cc.dvo.ru).

### 4.2. Construction of Recombinant Plasmids, Protein Expression and Purification

Expression plasmid encoding CGL mutants was constructed as described earlier [10] on the basis of pET40/CmAP plasmid which carried the gene of alkaline phosphatase CmAP as a reporter gene. CGL mutants were genetically engineered by oligonucleotide-specific mutagenesis approach. The amino acid substitutions were introduced into the forward and reverse gene-specific primers (Table 2).

The resultant mutant genes were amplified with the primers CGL-dir: 5′-AGCTGAGCTCGATGACGATGACAAGATGACAACGTTTCTTATCAAACACAAGGCCAGTG-3′ and CGL-rev: 5′-AGCTGTCGACTTAGGCATAAACTAAAACGCGCTTGTCTTT-3′, and ligated with the vector of pET-40b(+)/CmAP linearized by endonucleases SacI and SalI. The correct CGL cDNA sequence was verified by sequencing with ABI Prism Big Dye Terminator 3.1 Cycle Sequencing Kit and ABIPrism 310 Genetic Analyzer (Applied Biosystems, Foster City, CA, USA).

Recombinant lectins were expressed in *E. coli* Rosetta (DE3) and purified as described previously [10].

### 4.3. Lectin Activity Assay

The lectin activity assay was performed as described earlier (10). Briefly, 150 of porcine stomach mucin (PSM) with concentration of 0.1 mg/mL (0.1 M carbonate buffer, pH 9.5, containing 0.15 M NaCl) was added to each well of a polystyrene 96-well ELISA microtiter plate Maxisorp (Thermo Fisher Scientific, Waltham, MA, USA), incubated at 4 °C overnight, washed three times with the buffer containing 0.01 M Tris-HCl, pH 7.5, 0.15 M NaCl, 0.05% Triton X-100 (TBS-T) and three times with water. Bovine serum albumin (1 mg/mL) in TBS-T was added as described above. Samples containing recombinant CGL (0.2 mg/mL) were two-fold serially diluted in TBS-T and added in 150 mL aliquots to each well. The plate was incubated at room temperature for 1 h and then washed three times as described above. TBS-T was used as a negative control. Standard assay for alkaline phosphatase activity was carried out as described earlier [10]. One unit of AP activity was defined as the quantity of the enzyme required to release 1.0 μmol of p-nitrophenol from pNPP in 1 min. The specific activity was calculated as units per 1 mg of protein. All lectin activity assays were performed in three independent parallels for three to five times. Data were analyzed using the Student’s t-test of the SigmaPlot 2000 version 6.0 program (SPSS Inc.). Differences from controls were considered significant at *p* ≤ 0.05.

## 5. Conclusions

In this report we presented new details of structure-function relationships for a novel lectin from the mussel *C. grayanus*. In silico analysis of CGL complexes with galactose, globotriose and PSM-trisaccharide helped us to suggest the binding mechanisms of CGL. For the first time, it was shown that point mutation of residues that form hydrogen bonds with a terminal monosaccharide and not included in the conservative motif HPY(K)G, led to a change in the mucin-binding activity of mutants. The maximal decrease in the mucin-binding activity of the mutants Asn119Ala in Site 1 and Asn27Ala in Site 2 was due to the loss of all three hydrogen bonds with two terminal galactose residues of oligosaccharides in comparison with the wild type CGL. However, the efficiency of CGL binding depends on the composition of at least three terminal monosaccharide units in oligosaccharides. The amino acid residue Asp127 in Site 3 (and similar residues Asp35 and Asp83 in Sites 1 and 2) was found to play a decisive role in the higher lectin affinity to mucin due to forming an additional bond with the third fucose.

The ability of CGL to recognize Gb3 on the surface of breast cancer cells and bind mucin-type glycoproteins, which are often associated with oncogenic transformation, make it prospect in construction of a biosensor for cancer diagnostics. In this regard, the results elicited the individual contribution of His37, His129, Glu75, Asp127, His85, Asn27 and Asn119 amino acid residues from carbohydrate-binding sites to CGL activity could be helpful for designing an artificial analog of CGL with enhanced Gb3- and mucin-binding properties for applying in cancer diagnostics or anticancer therapy.

## Figures and Tables

**Table 1:** The mucin-binding activity of the recombinant CGL of wild and mutant types and the change in the binding energy (ΔE = Emut-Ewt) of the CGL mutants with galactose and globotriose.

Lectin	Gal Binding ΔE ^a^, kcal/mol	Globotriose Binding ΔE ^b^, kcal/mol	Mucin-Binding Activity ^c^, %
Asn119Ala *	1.9	4.9	9
Asn27Ala **	2.0	5.2	17
Asp127Ala ***	4.0	4.3	22
His85Ala **	3.6	3.4	20
Glu75Ala ***	3.4	4.7	31
His129Ala ***	3.5	3.6	43
His37Ala *	3.5	3.1	73

^a/b^—change in the binding energy of the CGL mutants with galactose (ΔE ^a^) or globotriose (ΔE ^b^); ^c^—mucin-binding activity of the wild type CGL was 100%; *—amino acid residues (aa) from Site 1, **—aa from Site 2, ***—aa from Site 3.

**Table 2:** Primers for construction of the recombinant plasmids.

Mutation	Sense Primer	Antisense Primer
Asn27Ala	5′–AGTAGCAACCCTGCTAACGCCACTAAGTTG−3′	5′–GCAGGACCAACTTAGTGGCGTTAGCAGGGT−3′
His37Ala	5′–GTCCTGCATAGCGATATCGCTGAAAGAATG−3′	5′–GGAAGTACATTCTTTCAGCGATATCGCTAT−3′
Glu 75Ala	5′–AGCTAATCCACCAAATGCCACCAATATGGTTC−3′	5′–TGATGCAGAACCATATTGGTGGCATTTGGTG−3′
His85Ala	5′–GTTCTGCATCAAGATCGTGCTGATCGGGCA−3′	5′–GAATAGTGCCCGATCAGCACGATCTTGAT–3′
Asn119Ala	5′–ATCCCCGAATCCACCGAATGCTACCGAAACAG−3′	5′–GTATAACTGTTTCGGTAGCATTCGGTGGAT−3′
Asp127Ala	5′–CAGTTATACATGGAGCTAAACATGCAGCCA−3′	5′−GAATTCCATGGCTGCATGTTTAGCTCCATGTA–3′
His129Ala	5′–ATACATGGAGATAAAGCTGCAGCCATGGAA−3′	5′–CAAAAATGAATTCCATGGCTGCAGCTTTATCT−3′
